# Origin and evolution of emerging *Liao ning Virus* (genus *Seadornavirus*, family *Reoviridae*)

**DOI:** 10.1186/s12985-020-01382-2

**Published:** 2020-07-14

**Authors:** Jun Zhang, Hong Liu, Jiahui Wang, Jiheng Wang, Jianming Zhang, Jiayue Wang, Xin Zhang, Hongfang Ji, Zhongfeng Ding, Han Xia, Chunyang Zhang, Qian Zhao, Guodong Liang

**Affiliations:** 1 Shandong Provincial Research Center for Bioinformatic Engineering and Technique, School of Life Sciences, Shandong University of Technology, Zibo, 255049 People’s Republic of China; 2 Key Laboratory of Special Pathogens and Biosafety, Wuhan Institute of Virology, Chinese Academy of Sciences, Wuhan, 430071 Hubei China; 3 State Key Laboratory of Infectious Disease Prevention and Control, National Institute for Viral Disease Control and Prevention, Chinese Center for Disease Control and Prevention, Beijing, 102206 People’s Republic of China

**Keywords:** *Liao ning virus*, LNV, *Seadornavirus*, Evolution, Migration

## Abstract

**Background:**

*Liao ning virus* (LNV) is a member of the genus *Seadornavirus*, family *Reoviridae* and has been isolated from kinds of vectors in Asia and Australia. However, there are no systematic studies describe the molecular genetic evolution and migration of LNVs. With the development of bioinformatics, viral genetic data combining the information of virus isolation time and locations could be integrated to infer the virus evolution and spread in nature.

**Methods:**

Here, a phylogenetic and phylogeographic analysis using Bayesian Markov chain Monte Carlo simulations was conducted on the LNVs isolated from a variety of vectors during 1990–2014 to identify the evolution and migration patterns of LNVs.

**Results:**

The results demonstrated that the LNV could be divided into 3 genotypes, of which genotype 1 mainly composed of LNVs isolated from Australia during 1990 to 2014 and the original LNV strain (LNV-NE97–31) isolated from Liaoning province in northern China in 1997, genotype 2 comprised of the isolates all from Xinjiang province in western China and genotype 3 consisted the isolates from Qinghai and Shanxi province of central China. LNVs emerged about 272 years ago and gradually evolved into three lineages in the order genotype 1, genotype 2 and genotype 3. Following phylogeographic analysis, it shows genotype 1 LNVs transmitted from Australia (113°E-153°E,10°S-42°S) to Liaoning province (118°E-125°E,38°N-43°N) in Northeast Asian continent then further spread across the central part of China to western China (75°E-95°E,35°N-50°N).

**Conclusion:**

LNVs were initially isolated from Liaoning province of China in the Northeast Asia, however, the present study revealed that LNVs were first appeared in Australia in the South Pacific region and transmitted to mainland China then rapidly spread across China and evolved three different genotypes. The above results suggested that LNV had the characteristics of long-distance transmission and there were great genetic diversity existed in the LNV population. Notably, current information of 80 strains of LNVs are limited. It is of great importance to strengthen the surveillance of LNVs to explore its real origin in nature and monitoring of the LNVs’ population variation and maintain vigilance to avoid LNV breaking through the species barrier and further clarify its relationship to human and animal infection.

## Introduction

*Liao ning virus* (LNV) belongs to the genus *Seadornavirus* of the family *Reoviridae* [1]. The genus *Seadornavirus* comprises 3 species, *Banna virus* (BAV), *Kadipiro virus* (KDV) and LNV [1–3]. The *Seadornaviruses* have a genome composed of 12 segments of double-stranded RNA, which decreases with the relative molecular weight during gel electrophoresis, and is named 1–12 segments [2]. The full length of the LNV genome is about 21,000 bp, with segment lengths that range from 3747 bp (segment 1) to 759 bp (segment 12). Each segment contains a open reading frame encodes a viral protein (Viral protein, VP), of which 5 are non-structural proteins (VP5, VP6, VP7, VP11, VP12), 7 are structural proteins (VP1, VP2, VP3, VP4, VP8, VP9, VP10).VP10 protein is an outer capsid structural protein which directly interacts with the receptor of host cells and is the main region that determines the antigenicity of the virus [3].

It is reported that the *Seadornaviruses* could be carried by a variety of blood-sucking insects including mosquitoes, ticks, and midges and even have been isolated from pigs, cattle and patients with fever and encephalitis [4–10]. Studies have also shown that BAV, which is the prototype of genus *Seadornavirus*, is an emerging pathogen that causes human viral encephalitis [2]. Therefore, *Seadornaviruses* may well be a group of newly discovered viruses close related to human and animal diseases [2, 4].

LNV was initially isolated from mosquitoes (*Aedes dorsalis*) in Liaoning province, northeast China in 1997 [10], since then additional isolates have been isolated from mosquitoes (genus *culex* and *Aedes*) in Shanxi province and Qinghai province in China [5, 7, 8].Interestingly,it is observed that all of the LNVs have only been isolated from the northern part of China and no LNV has been reported in other provinces in China and abroad [6].Once, the LNV has been considered to be a virus specie only restricted in northern part of China. Until a recent research reported that number of LNV strains have been isolated from mosquitoes of four genera (*Culex*, *Anopheles*, *Mansonia* and *Aedes*) in Australia in 2016 [11, 12]. This result demonstrated that LNV was not only limited in mainland China but also widely distributed in Australia in the South Pacific region. All of these research suggested that LNV was a widely distributed virus that could be transmitted by kinds of blood-sucking vectors and its geographical distribution even exceeds that of the other two *Seadornaviruses* (BAV, KDV) [1, 4].

Previous researches have reported on the molecular genetic evolution of LNV isolated in China and Australia [5, 7, 11]. However, the data used for analysis were only limited to the local LNV isolates [5, 7, 11]. China in Asia and Australia in the South Pacific are located in the northern and southern hemispheres, respectively. The natural environment in the two regions were significant different from each other. These observations raise several questions. What is the genetic relationship between the LNVs isolated from different regions? What are the characteristics of the molecular evolution of the entire LNV population? Where is the origin of LNVs? Recent revolutionary developments in virology bioinformatics provide unprecedented opportunities for analyzing the viral genetic data (nucleotide or amino acid) to model the evolutionary relationships between virus samples and help to explain epidemiological patterns and uncover processes of transmission [13–15]. Examples of such analyses include the estimation of origin and divergence time of *Japanese encephalitis virus* (JEV) [16, 17],identification of phylogenetic relationships among viral lineages in *Zika virus* (ZIKV) [18], inference of phylogeographic history and migration patterns of recent epidemics of *Middle East respiratory syndrome coronavirus* (MERS-CoV) [13] and *Ebola virus* (EBOV) [19]. Therefore, in the present study a comprehensive phylogeographic study of all the LNVs isolated worldwide were conducted to explore the molecular genetic evolution and migration patterns of LNVs isolated from different vectors since 1990 in China and Australia.

## Materials and methods

### Data set construction of the 10th segment gene of LNV

**Fig. 1:**
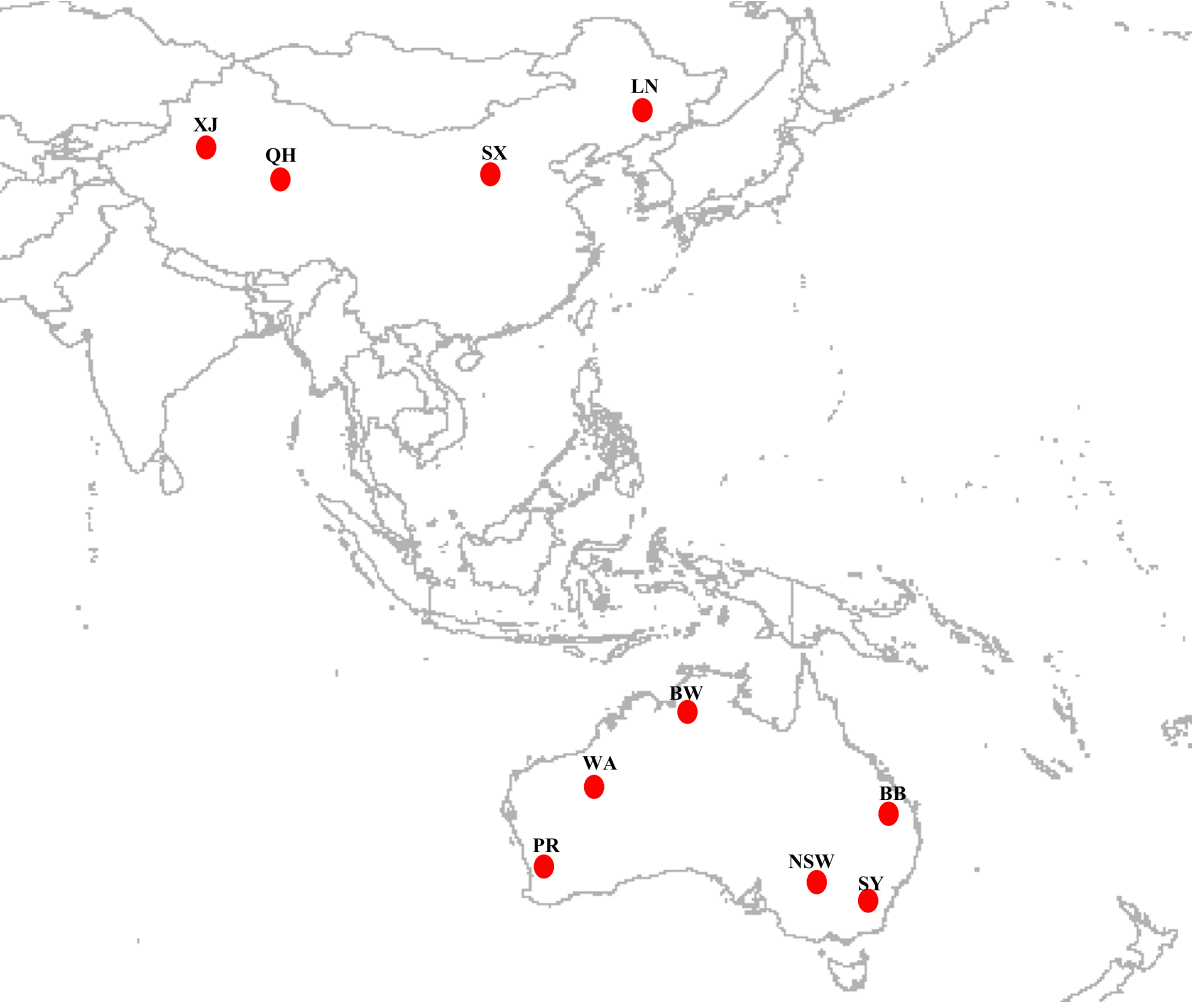
The distribution of LNV worldwide. LN: Liaoning province, SX: Shanxi province, QH: Qinghai province, XJ: Xinjiang province, WA: Western Australia, BB: Brisbane, SY: Sydney, NSW: New South Wales, BW: Bradshaw, PR: Peel region

LNV is a 12-segment double-stranded RNA virus. The 10th segment gene encodes the viral cell attachment protein, which directly interacts with the receptor on host cells [3]. Thus, the phylogenetic analysis based on this segment can best demonstrated the evolutionary and dispersal trends of viral strains from different isolation sites and vectors. Thus,we downloaded all the 10th segment gene sequences of LNV from GenBank as of June 2019 (Table 1). The data set contained 80 sequences, representing the samples isolated from kinds of mosquitoes:genus *Culex* (*n* = 64), genus *Aedes* (*n* = 10), genus *Ochlerotatus* (*n* = 3),unidentified mosquitoes (*n* = 3). The LNV isolation sites included the Liaoning province, Shanxi province, Qinghai province and Xinjiang province in China as well as New South Wales, Northern Territory, Queensland and Western Australia in Australia. The geographical distribution extends from Australia (113°E-153°E, 10°S-42°S) to mainland China (73°E-135°E, 3°N-53°N) (Fig. 1).

**Table 1:** The background information of LNVs used in this study

NO.	Strain	Location	Host	Date	Accession no.
1	LN-SW10194	Australia: Western Australia	mosquito	1990	MG725066.1
2	LNV-NE9712	China:Liaoning Province	*Aedes dorsalis mosquitoes*	1997	NC_007745.1
3	LNSV-NE97–31	China:Liaoning Province	*Aedes dorsalis mosquitoes*	1997	AY317108.1
4	LNV QLD2005	Australia: Brisbane	*Culex annulirostris*	2005	MG725847.1
5	J60	China:Xinjiang Province	*Culex mosquitoes*	2005	HM745532.1
6	J59	China:Xinjiang Province	*Culex mosquitoes*	2005	HM745531.1
7	J55	China:Xinjiang Province	*Culex mosquitoes*	2005	HM745530.1
8	J54	China:Xinjiang Province	*Culex mosquitoes*	2005	HM745529.1
9	J’49	China:Xinjiang Province	*Culex mosquitoes*	2005	HM745528.1
10	J’46	China:Xinjiang Province	*Culex mosquitoes*	2005	HM745527.1
11	J44	China:Xinjiang Province	*Culex mosquitoes*	2005	HM745525.1
12	J’44	China:Xinjiang Province	*Culex mosquitoes*	2005	HM745526.1
13	J’41	China:Xinjiang Province	*Culex mosquitoes*	2005	HM745524.1
14	J’37	China:Xinjiang Province	*Culex mosquitoes*	2005	HM745523.1
15	J’36	China:Xinjiang Province	*Culex mosquitoes*	2005	HM745522.1
16	J35	China:Xinjiang Province	*Culex mosquitoes*	2005	HM745521.1
17	J32	China:Xinjiang Province	*Culex mosquitoes*	2005	HM745520.1
18	J29	China:Xinjiang Province	*Culex mosquitoes*	2005	HM745519.1
19	J27	China:Xinjiang Province	*Culex mosquitoes*	2005	HM745518.1
20	J24	China:Xinjiang Province	*Culex mosquitoes*	2005	HM745517.1
21	J’18	China:Xinjiang Province	*Culex mosquitoes*	2005	HM745516.1
22	J’13	China:Xinjiang Province	*Culex mosquitoes*	2005	HM745515.1
23	J4	China:Xinjiang Province	*Culex mosquitoes*	2005	HM745514.1
24	J2	China:Xinjiang Province	*Culex mosquitoes*	2005	HM745513.1
25	J1	China:Xinjiang Province	*Culex mosquitoes*	2005	HM745512.1
26	B10	China:Xinjiang Province	*Culex mosquitoes*	2005	HM745511.1
27	B9	China:Xinjiang Province	*Culex mosquitoes*	2005	HM745510.1
28	B8	China:Xinjiang Province	*Culex mosquitoes*	2005	HM745509.1
29	B7	China:Xinjiang Province	*Culex mosquitoes*	2005	HM745508.1
30	B6	China:Xinjiang Province	*Culex mosquitoes*	2005	HM745507.1
31	B5	China:Xinjiang Province	*Culex mosquitoes*	2005	HM745506.1
32	XJ06121	China:Xinjiang Province	*Culex spp.*	2006	HM136838.1
33	XJ0615	China:Xinjiang Province	*Culex spp.*	2006	HM136869.1
34	XJ0642	China:Xinjiang Province	*Culex spp.*	2006	HM136867.1
35	XJ0640	China:Xinjiang Province	*Culex spp.*	2006	HM136866.1
36	XJ0656	China:Xinjiang Province	*Culex spp.*	2006	HM136863.1
37	XJ0672	China:Xinjiang Province	*Culex spp.*	2006	HM136862.1
38	XJ0610	China:Xinjiang Province	*Culex spp.*	2006	HM136860.1
39	XJ0635	China:Xinjiang Province	*Culex spp.*	2006	HM136859.1
40	XJ0621	China:Xinjiang Province	*Culex spp.*	2006	HM136858.1
41	XJ0626	China:Xinjiang Province	*Culex spp.*	2006	HM136857.1
42	XJ0623	China:Xinjiang Province	*Culex spp.*	2006	HM136854.1
43	XJ0603	China:Xinjiang Province	*Culex spp.*	2006	HM136853.1
44	XJ0658	China:Xinjiang Province	*Culex spp.*	2006	HM136852.1
45	XJ0670	China:Xinjiang Province	*Culex spp.*	2006	HM136851.1
46	XJ0624	China:Xinjiang Province	*Culex spp.*	2006	HM136850.1
47	XJ0659	China:Xinjiang Province	*Culex spp.*	2006	HM136849.1
48	XJ0665	China:Xinjiang Province	*Culex spp.*	2006	HM136846.1
49	XJ0654	China:Xinjiang Province	*Culex spp.*	2006	HM136845.1
50	XJ0632	China:Xinjiang Province	*Culex spp.*	2006	HM136844.1
51	XJ0611	China:Xinjiang Province	*Culex spp.*	2006	HM136843.1
52	XJ0627	China:Xinjiang Province	*Culex spp.*	2006	HM136842.1
53	XJ0653	China:Xinjiang Province	*Culex spp.*	2006	HM136841.1
54	XJ0620	China:Xinjiang Province	*Culex spp.*	2006	HM136840.1
55	XJ0657	China:Xinjiang Province	*Culex spp.*	2006	HM136837.1
56	XJ0661	China:Xinjiang Province	*Culex spp.*	2006	HM136836.1
57	XJ0606	China:Xinjiang Province	*Culex spp.*	2006	HM136835.1
58	XJ0648	China:Xinjiang Province	*Culex spp.*	2006	HM136834.1
59	XJ0614	China:Xinjiang Province	*Culex spp.*	2006	HM136833.1
60	XJ0662	China:Xinjiang Province	*Culex spp.*	2006	HM136832.1
61	XJ0616	China:Xinjiang Province	*Culex spp.*	2006	HM136831.1
62	XJ0602	China:Xinjiang Province	*Culex spp.*	2006	HM136830.1
63	XJ0737	China:Xinjiang Province	*Culex spp.*	2007	HM136848.1
64	XJ0735	China:Xinjiang Province	*Culex spp.*	2007	HM136847.1
65	XJ0719	China:Xinjiang Province	*Culex spp.*	2007	HM136856.1
66	XJ0727	China:Xinjiang Province	*Culex spp.*	2007	HM136855.1
67	XJ0740	China:Xinjiang Province	*Aedes dorsalis*	2007	HM136861.1
68	XJ0753	China:Xinjiang Province	*Aedes dorsalis*	2007	HM136865.1
69	XJ0746	China:Xinjiang Province	*Aedes dorsalis*	2007	HM136864.1
70	LNV NSW B115745	Australia: Sydney	*Ochlerotatus vigilax*	2007	MG725857.1
71	LN-5724	Australia: New South Wales	mosquito	2007	MG725042.1
72	LN-5798	Australia: New South Wales	mosquito	2007	MG725054.1
73	QH07130	China:Qinghai Province	*Culex modestus*	2007	HM230645.1
74	SX0794	China:Shanxi Province	*Aedes vexans*	2007	HM243144.1
75	SX0771	China:Shanxi Province	*Aedes vexans*	2007	HM243143.1
76	XJ0822	China:Xinjiang Province	*Aedes dorsalis*	2008	HM136870.1
77	XJ0833	China:Xinjiang Province	*Aedes dorsalis*	2008	HM136868.1
78	XJ0815	China:Xinjiang Province	*Aedes dorsalis*	2008	HM136839.1
79	LNV NT2013	Australia: Bradshaw	*Ochlerotatus vigilax*	2013	MG725842.1
80	LNV WA 60042	Australia: Peel region	*Ochlerotatus camptorhynchus*	2014	MG725869.1

### Time scaled phylogenetic and phylogeographic analysis of LNV

The 10th segment sequence database of LNV was analyzed using Bayesian Markov chain Monte Carlo (MCMC) method [20]. The GTR + I + G substitution model was selected to be the optimal model by MrModelTest [21]. Bayesian time scaled phylogenetic analysis and nucleotide substitution rate and most recent common ancestor (tMRCA) of LNVs were coestimated using the BEAST software package [22]. The relaxed clock model with different demographic models was tested [23], and the best models were selected by means of a Bayes factor (BF) test using marginal likelihoods values (2lnBF > 2) and 95% highest posterior density (HPD) intervals. The chain length was 1,000,000,000 generations. Convergence of parameters was checked using TRACER (http://beast.community/tracer) and was indicated as effective sample size (ESS>200), and the maximum clade credibility (MCC) tree was constructed using TreeAnnotator (http://beast.community/treeannotator) with 10% burn-in.

In order to clarify the geographical dispersal history of LNVs, the bayesian stochastic search variable selection (BSSVS) was used to provide evidence for statistically supported diffusion between state variables under BEAST software package [24]. This method estimates the most probable state at each node in the MCC trees, allowing us to reconstruct ancestral positions for ancestral viral lineages along the tree. For phylogeographic reconstructions, each region was coded as a discrete trait. BSSVS output and surfaces representing uncertainty for continuous diffusion processes were formatted as KML using the SPREAD software [25]. Determination of each locality was coordinated and performed using Google Earth. ArcGis was finally used to display the dispersal pattern of LNV based on the phylogeographic analysis.

### The sequence analysis of the 10th segment of LNV

Sequence similarity and nucleotide base composition analysis were performed using the CLUSTALX software [26], BioEdit software [27] MEGA-X [28] and MegAlign (DNASTAR, Madison, WI, USA). Hemi (version1.0) [29] software visualized the similarity comparison results of LNV and Rstudio made the nucleotide base composition analysis map of LNV.

### Three-dimensional structural analysis of VP10 of LNVs

The crystal structure of the cell attachment protein VP9 of BAV (PDB 1w9z) was selected as the best template for the homology modeling. The YASARA software [30], PyMol software [31] and VMD (version 1.9.2) [32] were used to analyze the structures and surface charge density of VP10 among LNV isolates.

## Result

### The evolution of LNV isolated worldwide based on the time-scaled phylogenetic analysis

**Fig. 2:**
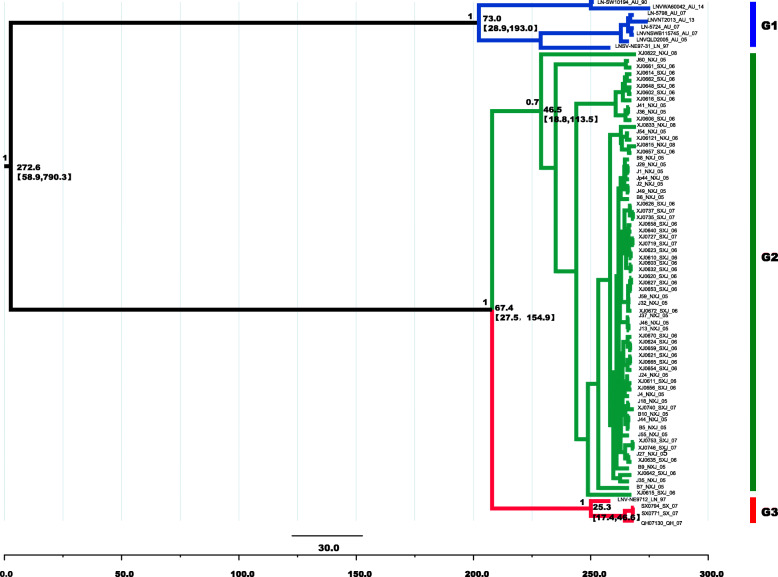
Evolution analysis of LNV isolated worldwide from 1990 to 2014. Maximum clade credibility tree for the 10th segment of LNVs isolated from 1990 to 2014 worldwide. The tree identified 3 distinct lineages, genotype 1, genotype 2 and genotype 3.Genotype 1 contained the initial LNV isolate (LNSV-NE97–31) and seven Australian isolates (Blue). Genotype 2 consisted of isolates all from Xingjiang province in western China (Green). Genotype 3 comprised LNV isolates from Qinghai province, Shanxi province and Liaoning province (another initial LNV isolate LNSV-NE97–12) (Red). Estimated tMRCAs of these lineages (with their 95% HPD values in parentheses) are presented. The posterior probability values of main branch nodes were indicated. The letters G1, G2 and G3 represent genotypes 1,2 and 3, respectively

According to the 95% HPD intervals and bayes factor, the Bayesian skyline model with a relaxed molecular clock was selected to be the best fit model. The MCC tree was established using the Bayesian Markov chain Monte Carlo (MCMC) approach (Fig. 2). The posterior probability values of each branch node were all greater than 0.7, showing the robustness of the result. The mean nucleotide substitution rate for the entire LNV population was estimated to be 1.6 × 10^− 3^ s/s/y (95%HPD:2.8 × 10^− 4^,3.3 × 10^− 3^) (Table 2). At this evolution rate, the tMRCA of the LNV was calculated to be 272.6 years ago (95%HPD:58.9,790.3). The LNV evolved chronologically into three major evolutionary populations, genotype 1, genotype 2 and genotype 3.Genotype1, which included the initial isolate (LNV-NE97–31) from Liaoning province of China and seven strains from Australian, emerged 73.0 years ago (95%HPD:28.9,193.0) and demonstrated to be the oldest lineage. Genotype 2 emerged about 46.5 years ago (95%HPD:18.8,113.5) and was composed of LNV strains isolated from Xinjiang province in western China. Genotype 3, which appeared approximately 25.3 years ago (95%HPD:17.4,46.6), included isolates from Qinghai province, Shanxi province and Liaoning province (another initial isolate LNV-NE97–12), and was the youngest LNV lineage.

**Table 2:** Sequence similarity and Bayesian Markov chain Monte Carlo analysis of LNVs

Genotypes	Nucleotide similarity		Amino acid similarity		tMRCA y (95% HPD)		Substitution rate s/s/y (95% HPD)
	Over all	Each group	Over all	Each group	Over all	Each group	Over all
1	70.4% ~ 100% (μ = 92.6%)	87.5% ~ 100% (μ = 94.3%)	75.0% ~ 100% (μ = 94.6%)	91.2% ~ 100% (μ = 96.4%)	272.6 (58.9,790.3)	73.0 (28.9,193.0)	1.6 × 10^−3^ (2.8 × 10^−4^,3.3 × 10^−3^)
2		92.5% ~ 100% (μ = 97.8%)		92.5% ~ 100% (μ = 98.9%)		46.5 (18.8,113.5)	
3		97.5% ~ 100% (μ = 99.2%)		98.1% ~ 100% (μ = 99.4%)		25.3 (17.4,46.6)	

Note: *μ* average nucleotide (amino acid) similarity, *y* years; *s/s/y* Substitutions per site per year

### Population dynamics of LNV

**Fig. 3:**
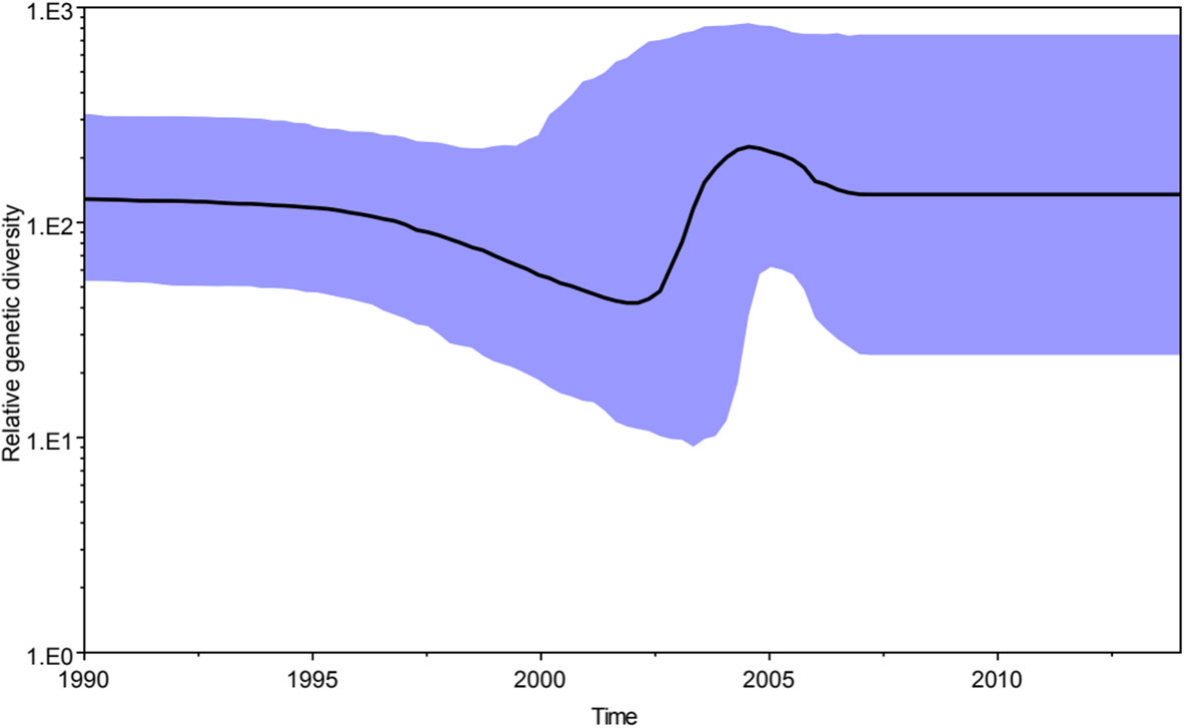
Bayesian skyline plot for LNV. Highlighted areas correspond to 95% HPD intervals

The skyline plot of the LNV population dynamics is shown in Fig. 3. The population dynamic of LNV kept stable from 1990 to 1997.Since 1997, the population diversity began to decrease and reached the lowest point at the year of 2003.From 2003 the LNV population diversity increased, peaking around 2005.After a minor decrease during around 2005 to 2006, the virus population was stable from 2006 to 2014.

### The sequence similarity analysis of LNV

**Fig. 4:**
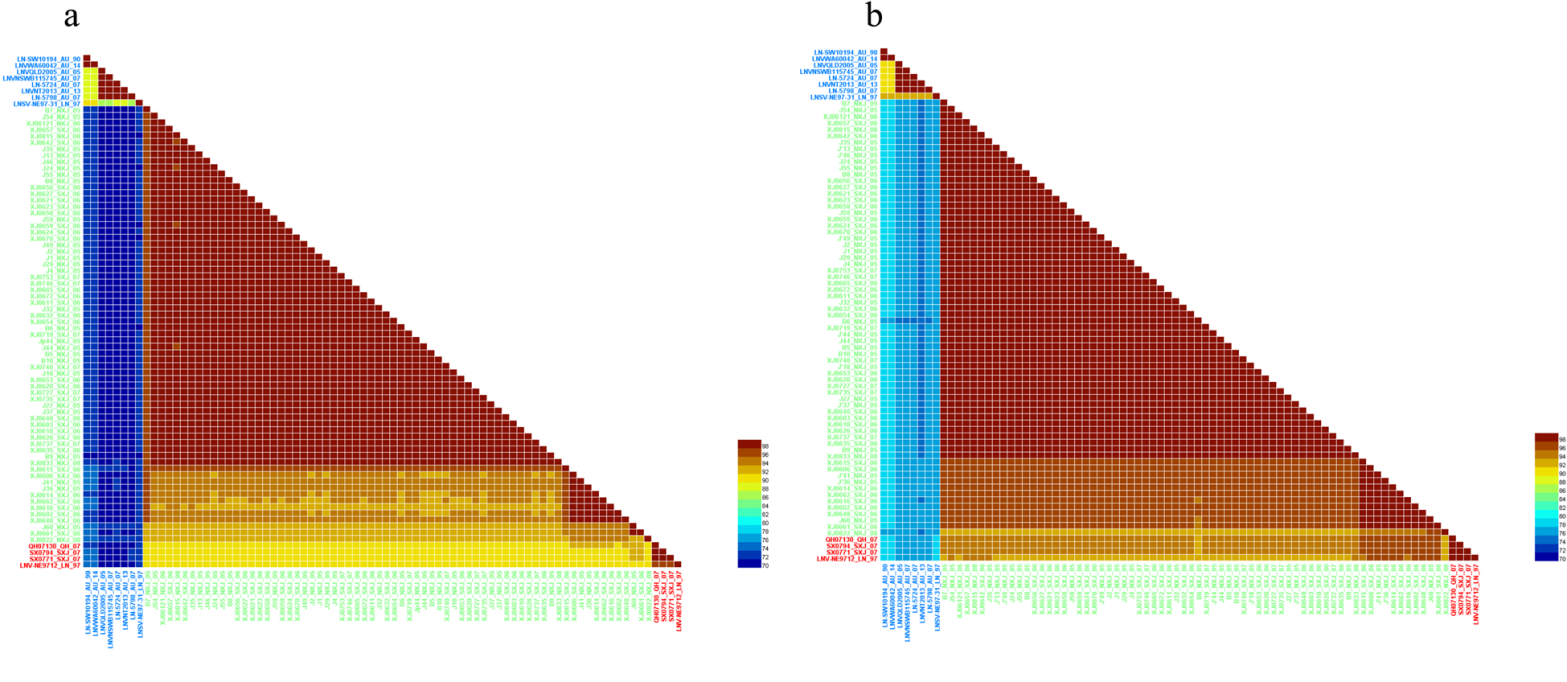
The Nucleotide and amino acid similarity of the 10th segment of LNV. **a**: Nucleotide similarity of LNV. **b**: Amino acid similarity of LNV. The horizontal and vertical axes represent the names of different genotypes of LNV isolates. The names of the isolates colored blue, green and red represent the genotypes 1,2 and 3, respectively

The Sequence analyses of 80 strains of LNV isolated from different vectors and locations during 1990–2014 revealed that the level of nucleotide similarity varied from 70.4 to 100% with an average value of μ = 92.6% while the amino acid similarity ranged from 75.0 to 100% with an average value μ = 94.6% (Fig. 4). Genotype 1 exhibited nucleotide similarity between 87.5–100% (μ = 94.3%) and amino acid similarity range from 91.2 to 100% (μ = 96.4%).Genotype 2 exhibited nucleotide similarity from 92.5 to 100% (μ = 97.8%) and amino acid similarity from 92.5 to 100% (μ = 98.9%). Genotype3 exhibited nucleotide similarity from 97.5 to 100% (μ = 99.2%) and amino acid similarity from 98.1 to 100% (μ = 99.4%). The results demonstrated that the genotype 1 population possessed the highest degree of nucleotide and amino acid variation among all the three LNV genotypes.

### Nucleotide composition of the 10th segment genome of LNV

**Fig. 5:**
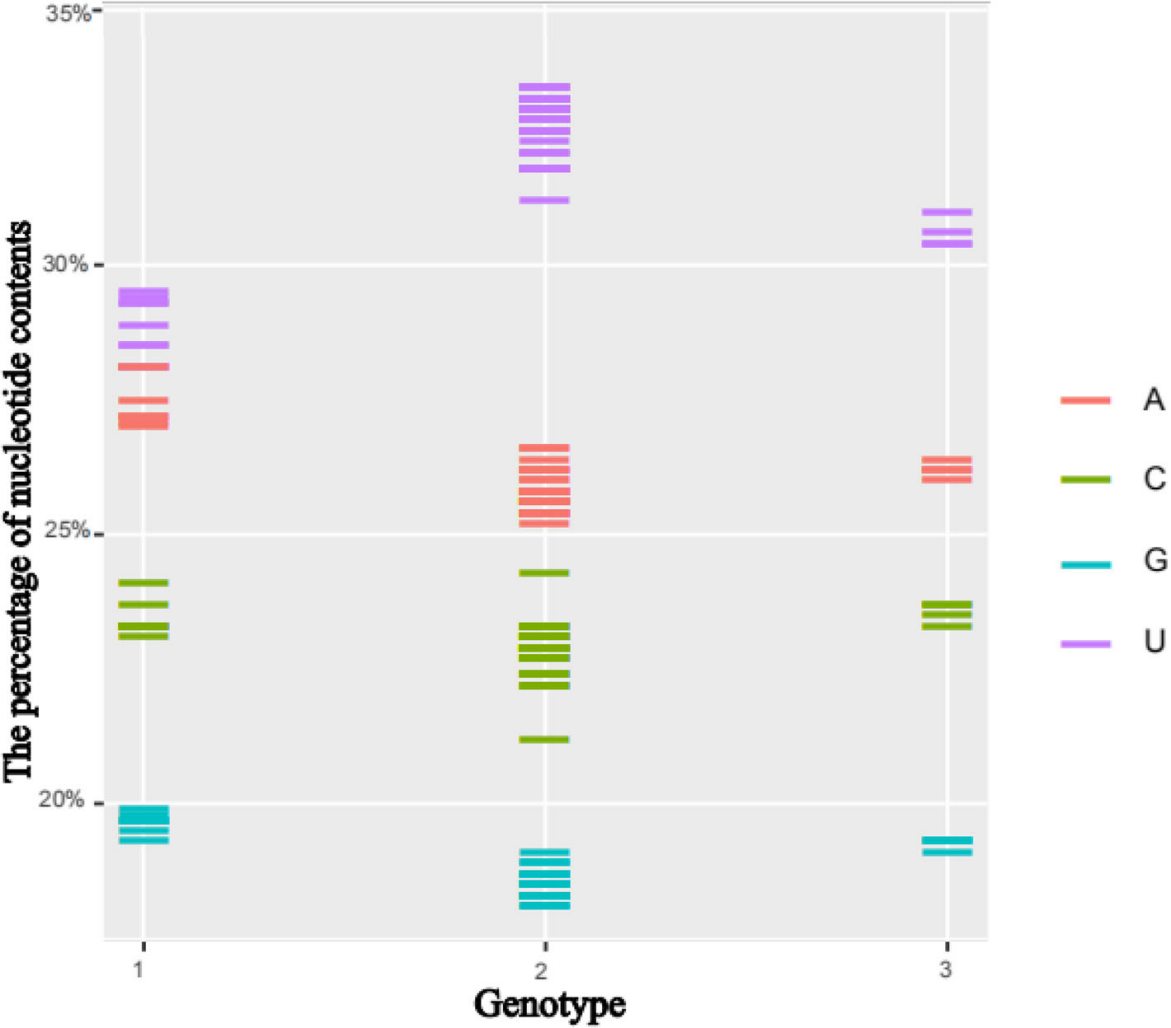
The A, U, G and C contents of different genotypes of the 10th segment of LNV. The vertical axes indicates the percentage of nucleotide contents. The cross bar represents the different isolates in the same genotype. The red bar identifies adenine (A), green identifies cytosine (C), cyan identifies guanine (G) and violet identifies uracil (U)

The values of nucleotide contents in the entire LNV population and each genotype were analyzed (Fig. 5). The results showed that VP10 genome of LNV was rich in A and U (A + U > C + G) and the percentage of AU was reaching 59.5%. The A%, U%, G%, C% are 26.0% ±0.6 (mean ± SD), 32.3% ± 1.3, 18.7% ± 0.4, 23.1% ± 0.4, respectively. There was a common trend in the usage of A,U,G,C between the three genotypes that was U > A > 0.25 > C > G (Fig. 5). As for each genotype, the average contents of A,U,G,C for genotype 1 were 27.6% ± 0.5, 29.0% ± 0.5, 19.8% ± 0.2, 23.5% ± 0.3,for genotypes 2 were 25.8% ± 0.3, 32.8% ± 0.5,18.5% ± 0.2, 23.0% ± 0.4 and these values for genotypes 3 were 26.3% ± 0.2, 30.7% ± 0.3, 19.4% ± 0.1, 23.7% ± 0.2. The value of the A content in genotype 1 was the highest while that in genotype 2 was the lowest.

### The dispersal route of LNV base on phylogeographic analysis

**Fig. 6:**
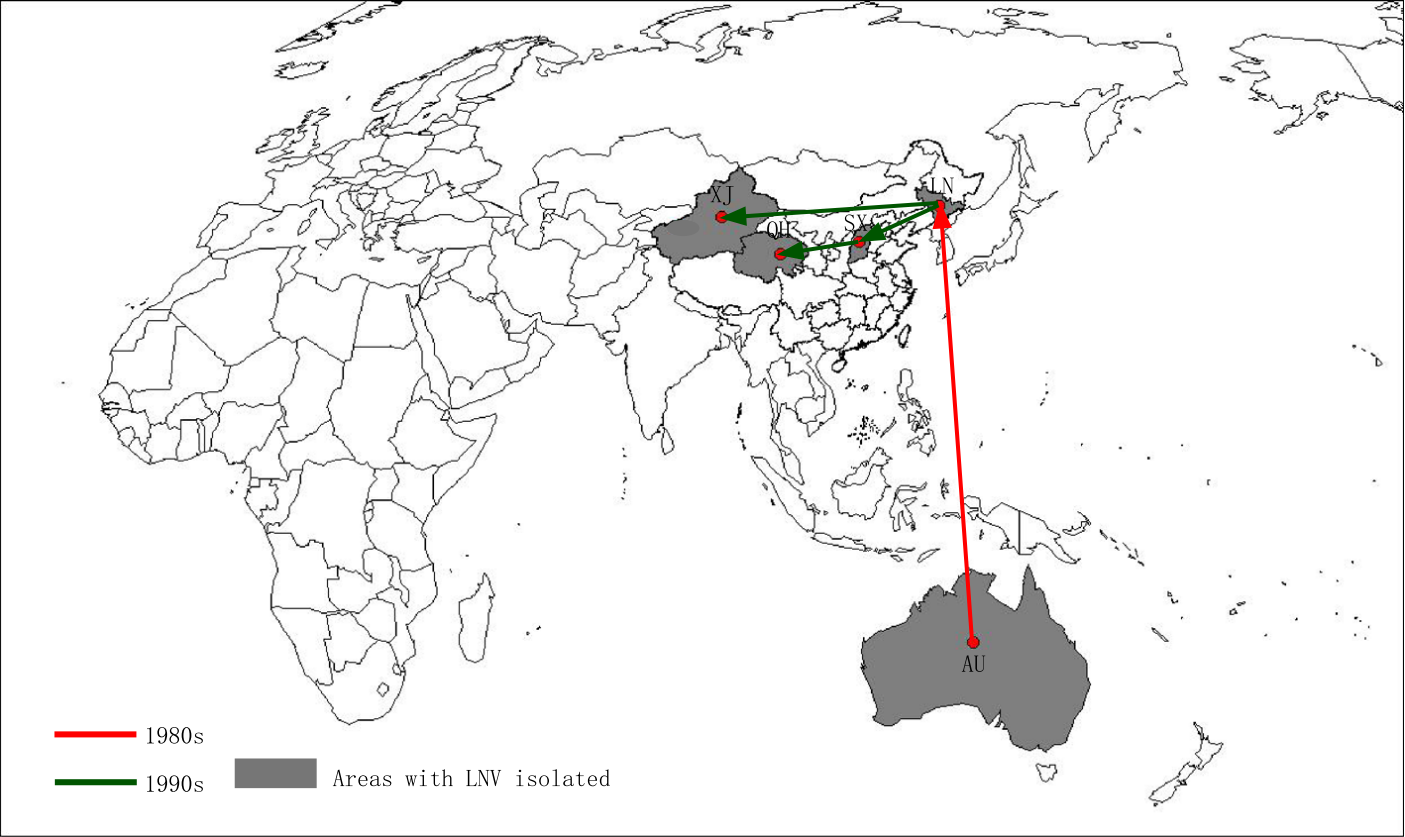
Dispersal route of LNVs based on phylogeographic analysis. Gray shadow presents the areas with LNV isolated. Line and arrow represents the transmission route and direction of LNV. Red line: Spread from Australia to Liaoning province of China in 1980s; Green line: Spread from Liaoning province to Shanxi province, Qinghai province and Xinjiang province in 1990s. LN: Liaoning province, SX: Shanxi province, QH: Qinghai province, XJ: Xinjiang province, AU: Australia

The estimated history of LNV dispersal route over time is shown in detail in Fig. 6. According to our results of the phylogeographic analysis, LNV was originated in Australia in the south pacific region and then initially introduced to Liaoning province in China in the Northeast Asia around 1980s.Subsequently, the virus was further westwarded spread to Shanxi province, Qinghai province and Xingjiang province of China during 1990s.

### The amino acid comparison and the three dimensional structural analysis of the cell attachment protein (VP10) of LNV

To characterize the mutations in the cell attachment protein (VP10) between the three genotypes of LNV, we analyzed the amino acids encoded by the VP10 genes derived from the previously mentioned LNV isolates. The results revealed that the ORF of VP10 was 753 nucleotides in length and encoded 250 amino acids. The amino acid sequences of genotype 1 were significantly different from those of the rest two genotypes while the genotype 2 and 3 shared a great similarity in amino acid sequences. The detailed results were that a total of 20 common amino acid differences identified between genotype 1 to genotype 2 and 3 (Table 3). The amino acid sequences were highly similar between genotype2 and 3 and there were only 3 amino acid differences were identified (Table 3). When compared the amino acid sequences of genotype 1 to those of genotype 2 and 3, twenty common amino acid differences were identified as follows: S44A, A60G, D64G, S65N, H68V, N82A, P99A, V122T, M131S, A132G, S160N, I167S, Q171S, A181L, E207D, S209V, T217N, S219N, N223H, N238S.The amino acid sequences of genotype 2 and 3 were highly similar, there were only three amino acid mutations: S58A, A130N, L210H.

**Table 3:** Comparison of VP10 amino acid sequences between different LNV genotypes

Genotypes	Positions of amino acid																						
	44	58	60	64	65	68	82	99	122	130	131	132	160	167	171	181	207	209	210	217	219	223	238
1	S	A	A	D	S	H	N	P	V	N	M	A	S	I	Q	A	E	S	Q	T	S	N	N
2	A	S	G	G	N	V	A	A	T	A	S	G	N	S	S	L	D	V	L	N	N	H	S
3	A	A	G	G	N	V	A	A	T	N	S	G	N	S	S	L	D	V	H	N	N	H	S

Note: Only the different amino acids among three genotypes of LNV are shown in the table

**Fig. 7:**
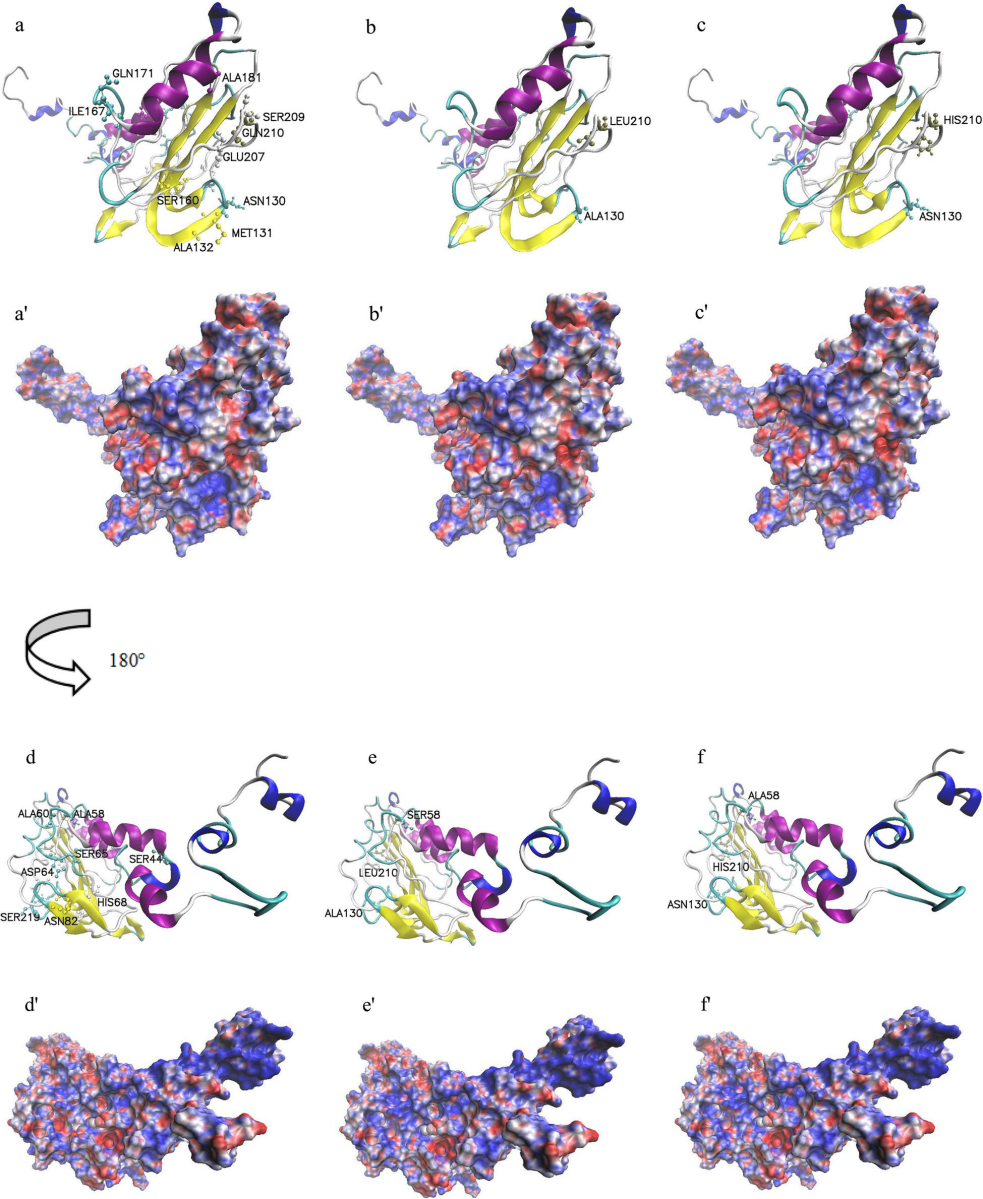
The theoretical three-dimensional structure and surface charge of different genotypes of LNV. **a**, **b**, **c**: The theoretical three-dimensional structure of LNV of genotype1,2,3 and the differences sites of amino acid within 3 genotypes. **a**’, **b**’, **c**’: The theoretical three-dimensional structure as well as the surface charge distribution of LNV of genotype 1,2,3. def and d’e’f’ are the angles of view of abc and a’b’c’ rotated the Y axis by 180 degrees, respectively

In order to further explore whether these mutations affected the three shape and the surface charge density of the cell attachment protein of LNV, the three dimensional structures and electrostatic potential analysis have been conducted between different genotypes of LNV. Seven of the twenty common amino acid differences were located in the β-sheet (residues 82, 122, 131, 132, 181, 223, and 238), while the remaining thirteen were located in the loop region (residues 44, 60, 64, 65, 68, 99, 160, 167, 171, 207, 209, 217 and 219). These differences have some effect on the structure and electrostatic properties of VP10 between the different genotypes. In particular, the residue at position 64, 65, 131, 132, 167, 171, 207, 209 and 219.There were great differences existed the R group of the amino acid and the electrical polarity of the mentioned 9 mutations, resulting in significant differences in structure and charge at these sites between the LNVs (Fig. 7a’, b’, c’).

## Discussion

During the arboviruses survey in China in 1997, two virus strains (LNSV-NE9731 and LNV-NE9712) were obtained from *Aedes dorsalis* mosquitoes in Liaoning province in North-East of China which were found to cause cytopathic effect in C6/36 cells. The virus was designated *Liao ning virus* (LNV) [3, 10]. Since then, additional strains of LNV were isolated from Shanxi province, Qinhai province and Xinjiang province [7, 8]. A more than 30 years national arbovirus surveillance in mainland China revealed the LNV strains had only been isolated in a long and narrow region covering 73–125 ° E and 31–48 ° N in the northwest to northeast part of the country. No strains of LNV have been reported isolated in other regions of China or abroad [6]. However, a recent research reported a total of 35 strains of LNV were isolated from mosquitoes belonging to four genera (*Culex*, *Anopheles, Mansonia and Aedes*) collected from 1988 to 2014 in Australia in the southern hemisphere. The isolation sites included New South Wales, Northern Territory, Queensland and Western Australia, almost across the entire Australian continent. Thus, it is obvious that the geographical distribution and the vectors of LNV are wide and variable in Australia. What is more, the initial LNV isolate in Australian was obtained from mosquitoes collected in 1988, predating the first Chinese LNV isolate which was obtained in 1997 [10, 11].

Several phylogenetic analysis have been conducted on the LNV’s nucleotide sequences, previously. The LNVs isolated in China could be divided into 3 evolutionary branches. The LNSV-NE9731 strain isolated in Liaoning province in 1997 formed an independent evolutionary branch while another Liaoning isolates named LNSV-NE9712 clustered together with the isolates from Qinghai province and Shanxi province that formed a branch. All of the Xinjiang isolates grouped together formed the largest evolutionary branch [5, 33]. All of the Australian LNV isolates were divided into two disparate lineage, one composed the isolates from eastern and northern Australia and the other included the isolates from western and southern Australia [11, 12]. In this study, a comprehensive molecular phylogenetic analysis of all the LNVs isolated from China and Austrila demonstrated that the LNVs could be divide into three genotypes (Fig. 2). Genotype 1 included LNSV-NE9731 (isolated in Liaoning provinve of China in 1997) and 7 strains from Austrila (the mosquito collection time was 1990,2005,2007,2013 and 2014, respectively). A total of 68 strains of LNV isolated from Xinjiang province of western China clustered together that formed an independent branch. And the strain LNV-NE9712 isolated in 1997 together with the Qinghai and Shanxi isolates formed the genotype 3 evolutionary branch. This result suggested that the Australia LNV isolates were not only restricted and circulated in Australia and even this virus population transmitted a long way to northern China in Asia then evolved into new LNV populations with local circulation characteristics.

The time-scaled evolutionary analysis of LNV revealed the branching of the lineages occurred in the following order:genotype 1 emerged 73 years ago (95%HPD:28.9, 193.0), the genotype 2 at 46.5 years ago (95%HPD:18.8, 113.5) and genotype 3 at 25.3 years ago (95%HPD:17.4, 46.6.) Thus, genotype 1 is the oldest lineage and the genotype 3 is the youngest one (Table 2). The current results lead to an estimate that the most recent common ancestor of LNV appeared about 277 years ago which similar to that of another member of *seadornaviruses* BAV, whose tMRCA is 105 years ago [34], indicating that LNV is an emerging virus population.

Although the LNVs were initially isolated from several inland provinces in China, the phylogeopgraphic analysis of our study showed that the LNV likely originated from Australia in the South Pacific region and the genotype 1 viruses spread northward from Australia to Liaoning province in northeast China. The reasons that LNV originated in Australia and spread to the Asian continent may be related to the following factors: First, Australia is located in the South Pacific region and contains a variety of geographic climate types, including the subtropical humid climate in the east, the savanna climate in the northwest, the tropical rain forest climate in the northeast, and Mediterranean climate in the southwest [35]. The climate types in Australia facilitate the local host vectors diverse and can breed LNV population with a strong adaptability. Second, the nucleotide similarity of genotype 1 was the most divergent between all of the three genotypes, indicating greater population variability exists in Australian LNVs.

Additionally, there was a very interesting finding that the AU content was relative high in VP10 of LNV, whereas the AU content was also high within the LNV’s whole viral genome (data not shown). These results were consistent with the prior studies wherein A and U frequencies were higher than C and G frequencies for avian *rotaviruses* and some *flaviviruses* including *dengue virus* (DENV), *West Nile virus* (WNV), y*ellow fever virus* (YFV) and JEV [36–39]. Besides, the nucleotide composition of the BAV, which is the prototype species of genus *Seadornavirus*, was also observed a higher AU content [40]. It has been reported that the AU-rich genome structure facilitate the *Human Immunodeficiency virus* (HIV) to avoid recognition by the innate immune system of host cell [41]. However, the biological causes and the consequence for increased A and U within the LNV and other mentioned arboviruses genome are still unknown, so enhanced experimental studies are required to explore the AU rich molecular function in LNV.

This may be the reasons why LNVs have been continuously isolated from 12 species of mosquitoes belonging to four genera in different geographical and climatic regions in Australia, during the last 30 years [11, 12]. However, in China the LNV has only been isolated from a long and narrow region restricted from 31°N to 48°N, where belongs to the north temperate zone with cold climate, less rain and low species diversity [5, 7, 10]. What is more, after transmission from Australia to China, the genotype 1 LNV population gradually adapted to the local natural environment and evolved new strains with regional genetic characteristics. The newly evolved LNVs (genotypes 2 and 3) contain 20 amino acid mutations compared with the initial LNVs (genotype 1) in the cell attachment protein (VP10), which altered the structure and electrostatic presentation influencing the binding properties to host vectors. This might be one of the reasons why the newly evolved LNVs (genotyp 2 and 3) were restricted to a relative narrow range of vectors and habitat. Compared with genotype 1 and genotype 2, the genotype 3 which located in the central part of China contains 2 unique amino acids, which were identical with genotype 1 but different from genotype 2,indicating that this lineage is at evolutionary transitional position which preserved the genetic information of the original LNV population and also evolved novel genetic information sites during spread to new locations. When compared with original LNV population, the genotype 2 LNVs in Xinjiang province contained the the maximum numbers of amino acid mutations thus it was the the most divergent lineage and formed a independed evolutionary branch. The genetic informative sites of the entire LNVs population confirmed its transmission path from north to south, and then from east to west.

## Conclusions

In this study, the evolution analysis of LNVs revealed that the virus belongs to the emerging virus group. In particular, it was suggested that the genotype 1 LNVs were a group of segmented double-strand RNA virus with extremely adaptability, which have a large group of vectors, a high rate of genetic variation and apparent active transmissibility that can adapt to different geographical environments over a quite long distance. Recently, the genome information of LNV was reported to be detected in *Aedes aegypti* of African origin, reminding us that LNVs was not only limited to China in Asia and Australia in the South Pacific region but may well be extended to Africa and even posing a high risk of spreading to new areas such as Central Asia and Europe. It is well known that genetic mutation such as recombination or reassortment can easily occur in segmented RNA virus. For example, BAV which also belongs to the *Seadornavirus*, was originally discovered in southern China. However, several novel variants of BAV have been found, such as the BALV isolate from Hungary [33] and the *Mangshi virus* from southern China [42]. Considering LNV was the only specie in *Seadornavirus* that can replicate in mammalian cell lines and cause fatal haemorrhagic symptoms in mice [3], it might be a pathogen that has great potential to cause disease in human and (or) animals. Therefore, to strengthen the research on genetic variation of LNV and to clarify the relationship between LNV and zoonosis is not only a research topic for virologists, but also a scientific issues for public health communities.

## Data Availability

All data and materials are included in this published article.

## Abbreviations

LNV: *Liao ning virus*
LNVs: *Liao ning viruses*
BAV: *Banna virus*
KDV: *Kadipiro virus*
RNA: RibonucleicAcid
bp: basepair
VP: Viral protein
VP10: The tenth segnment of viral protein
MCMC: The Bayesian Markov chain Monte Carlo
tMRCA: The time of most recent ancestor
BEAST: Bayesian Evolutionary Analysis Sampling Trees
BF: Bayes factor
HPD: Highest posterior density
MCC: The maximum clade credibility
ESS: The effective sample size
BSSVS: The bayesian stochastic search variable selection
KML: Keyhole Markup Language
SPREAD: Spatial phylogenetic reconstruction of evolutionary dynamics
SD: Standard Deviation
A,U,G,C: Adenine,Uracil,Guanine,Cytosine
ORF: Open reading frame
BALV: *Banna-like virus*
